# Activation of Sphingosine 1-Phosphate Receptor 1 Enhances Hippocampus Neurogenesis in a Rat Model of Traumatic Brain Injury: An Involvement of MEK/Erk Signaling Pathway

**DOI:** 10.1155/2016/8072156

**Published:** 2016-11-29

**Authors:** Yuqin Ye, Zhenyu Zhao, Hongyu Xu, Xin Zhang, Xinhong Su, Yongxiang Yang, Xinguang Yu, Xiaosheng He

**Affiliations:** ^1^Department of Neurosurgery, Xijing Hospital, Fourth Military Medical University, Xi'an 710032, China; ^2^Department of Neurosurgery, PLA 163rd Hospital (Second Affiliated Hospital of Hunan Normal University), Changsha 410000, China; ^3^Department of Neurosurgery, PLA General Hospital, Beijing 100853, China

## Abstract

Among sphingosine 1-phosphate receptors (S1PRs) family, S1PR1 has been shown to be the most highly expressed subtype in neural stem cells (NSCs) and plays a crucial role in the migratory property of NSCs. Recent studies suggested that S1PR1 was expressed abundantly in the hippocampus, a specific neurogenic region in rodent brain for endogenous neurogenesis throughout life. However, the potential association between S1PR1 and neurogenesis in hippocampus following traumatic brain injury (TBI) remains unknown. In this study, the changes of hippocampal S1PR1 expression after TBI and their effects on neurogenesis and neurocognitive function were investigated, focusing on particularly the extracellular signal-regulated kinase (Erk) signaling pathway which had been found to regulate multiple properties of NSCs. The results showed that a marked upregulation of S1PR1 occurred with a peak at 7 days after trauma, revealing an enhancement of proliferation and neuronal differentiation of NSCs in hippocampus due to S1PR1 activation. More importantly, it was suggested that mitogen-activated protein kinase-Erk kinase (MEK)/Erk cascade was required for S1PR1-meidated neurogenesis and neurocognitive recovery following TBI. This study lays a preliminary foundation for future research on promoting hippocampal neurogenesis and improving TBI outcome.

## 1. Introduction

Traumatic brain injury (TBI) is a commonly-seen cause of brain damage and often results in a series of nonreversible neuronal loss and neurological deficits [1, 2]. Neural stem cells (NSCs), located in the subgranular zone (SGZ) of hippocampal dentate gyrus (DG), are capable of proliferating, differentiating, and integrating into the existing neuronal circuits that play a pivotal role in neurogenesis throughout life in mammalian brain [3, 4]. These self-renewing cells contribute not only to embryonic brain development, but also to neural regeneration after TBI in adults [3, 5, 6]. Endogenous neural regeneration in hippocampus represents a special type of neural plasticity that has huge potential to replenish neural loss and restore neurological function after TBI [5]. However, severe and permanent functional disability in TBI often occurred due to limited endogenous neurogenesis capacity of adult brain [4, 7]. Therefore, activating endogenous NSCs to enhance neurogenesis in hippocampus is considered to be one of the promising strategies for TBI rehabilitation. As a matter of fact, the details of NSCs proliferation and differentiation remain not to be fully elucidated since there are a variety of complex regulatory factors found to be involved in endogenous neurogenesis process [3, 8]. Hence, identification of these unknown factors would help us understand more about hippocampal neurogenesis and provide new clues in order to improve neural repair after TBI.

As classical G-protein-coupled receptors, sphingosine 1-phosphate receptors (S1PRs) are a critical immune-modulatory receptor family that consists of S1PR1, S1PR2, S1PR3, S1PR4, and S1PR5 [9]. S1PRs family is enriched in central nervous system (CNS) and recognizes lots of bioactive signaling ligands including lipid mediator sphingosine 1-phosphate (S1P) to regulate neuronal survival, gliosis, astrocyte migration, and other biological processes in both physiological and pathological circumstances [10–12]. Of the five known S1PRs, S1PR1 expressed in hippocampal primordium and subventricular zone (SVZ) could be detected as early as E14 during CNS development [13]. In particular, increasing evidence suggested that NSCs harvested all subtypes of S1PRs, in which S1PR1 was the most highly expressed [14, 15]. And S1PR1 might be a presumed S1PRs subtype responsible for the proliferation and morphological changes of NSCs induced by S1P* in vitro* [16]. Recently, it has been demonstrated that S1PR1 plays an important role in the transplanted NSCs migration toward injured area of spinal cord for rebuilding [15]. However, little is known about the potential* in vivo* effect of S1PR1 on the proliferation and differentiation of NSCs in hippocampus following TBI. The mitogen-activated protein kinases (MAPK)/extracellular signal-regulated kinase (Erk) signaling pathway is a pivotal cell cascade that takes a crucial role in multiple factors involved in hippocampal neurogenesis [16–18]. Till today, whether and how MAPK-Erk kinase (MEK)/Erk cascade is implicated in the presumed S1PR1-assicoated neurogenesis after TBI remain poorly understood. The present study aims to clarify the potential role of S1PR1 in hippocampal NSCs proliferation and differentiation in response to TBI, and its underlying link with MEK/Erk cascade.

## 2. Materials and Methods

### 2.1. Animals and Experimental Groups

Healthy male Sprague-Dawley rats (weighing 250–300 g) were provided by Laboratory Animal Center of Fourth Military Medical University. The animals were maintained under an environment with 24–27°C, 60% humidity, a 12-hour light/dark cycle (light on from 07:00 to 19:00), and enough food and water. All the experimental procedures were carried out in accordance with the National Experimental Animals Guidelines and approved by the Institutional Animal Care and Use Committee of Fourth Military Medical University, Xi'an, China. All measures were taken to minimize animal suffering.

One hundred and sixty-five rats were randomly assigned to eight groups as below: sham group (*n* = 15), TBI group (*n* = 35), TBI-treated with vehicle 1% dimethyl sulfoxide (DMSO) group (TBI+Vehicle group, *n* = 23), TBI-treated with selective S1PR1 agonist SEW2871 group (TBI+SEW group, *n* = 23), TBI-treated with SEW2871 and S1PR1 antagonist VPC23019 group (TBI+SEW+VPC group, *n* = 18), TBI-treated with SEW2871 and MEK/Erk inhibitor U0126 (TBI+SEW+U0126 group, *n* = 17), TBI-treated with VPC23019 (TBI+VPC group, *n* = 17), and TBI-treated with MEK/Erk activator erucin (ERN) and VPC23019 (TBI+VPC+ERN group, *n* = 17).

### 2.2. TBI Model Establishment

TBI was induced by a controlled cortical impact (CCI) device (Hatteras Instruments, Cary, NC, USA). All animals were anesthetized by intraperitoneal (i.p.) injection of sodium pentobarbital (60 mg/kg) and were placed on the stereotaxic frame (Kopf Instruments, Tujunga, CA, USA) of injury device. The rat head was horizontally secured by two lateral ear pins and an incisor bar. After a sagittal incision was performed on the scalp, a 4.0 mm diameter craniotomy was made between lambda and bregma sutures and 3.0 mm lateral to the sagittal suture on the right. The skull flap was removed to expose the dura, on which a perpendicular impact was performed by a piston rod with its contact surface of 3.0 mm diameter. The brain injury was conducted in accordance with the following biomechanical parameters: 1.5 mm for vertical dura shift, 100.0 ms for contact time, and 3.0 m/s for piston velocity. Then, the skull flap was restored and the scalp was sutured. Rats in sham surgery group were subjected only to craniotomy without cortical impact. During the surgical procedure and recovery period, a heating pad was used to keep the rat body temperature at 36.0–37.0°C. All TBI rats appeared with arched back, erect hair, unconsciousness, and slow respiration but recovered to normal within the following 2 hours. No rats died in all groups.

### 2.3. Drugs and 5-Bromo-2-deoxyuridine (BrdU) Administration

**Figure 1:**
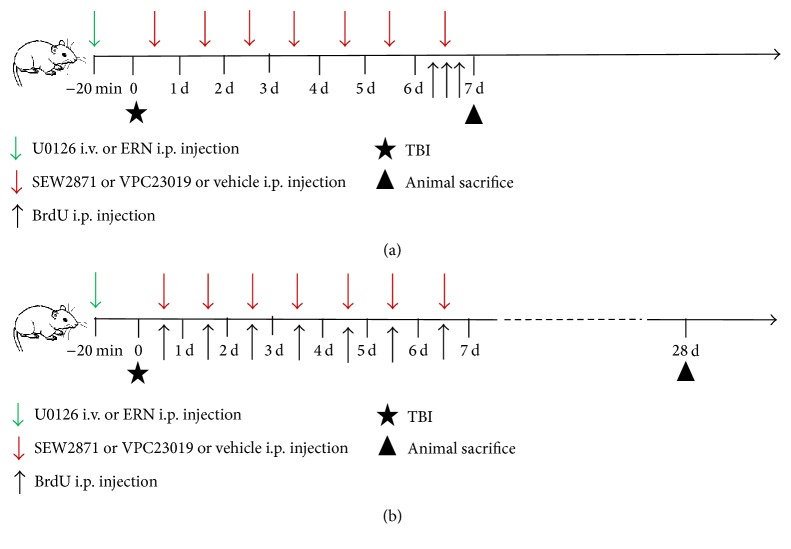
Schematic diagram of drugs and 5-bromo-2-deoxyuridine (BrdU) administration. The time-point of traumatic brain injury (TBI) model establishment was defined as zero point (indicated by black pentagram). (a) For the assay of neural stem cells (NSCs) proliferation, BrdU (100 mg/kg) was injected intraperitoneally (i.p.) three times with 8-hour interval at 6 days after TBI (indicated by black arrow) and the animals were sacrificed for perfusion at 7 days after TBI (indicated by black triangle). (b) For the analysis of NSCs differentiation, BrdU (100 mg/kg) was injected i.p. seven times with 24-hour interval 1–7 days after TBI (indicated by black arrow) and the animals were sacrificed for perfusion at 28 days after TBI (indicated by black triangle). SEW2871 (1.0 mg/kg/day) or VPC23019 (0.5 mg/kg/day) or vehicle (0.5 mL/kg/day) was administrated i.p. in rats of the corresponding group seven times within 24-hour interval 1–7 days after TBI (indicated by red arrow in (a) and (b)). Erucin (ERN, 12.5 mg/kg) or U0126 (0.2 mg/kg) was administrated i.p. or intravenously (i.v.) in rats of TBI+SEW+U0126 group 20 minutes before TBI (indicated by green arrow in (a) and (b)).

For administration of S1PR1 agonist or antagonist (Figures 1(a) and 1(b)), SEW2871 (Cayman Chemical, 10006440, Ann Arbor, MI, USA) and VPC23019 (Cayman Chemical, 13240, Ann Arbor, MI, USA) were, respectively, dissolved in DMSO (5% DMSO in 0.05 M PBS), making each individual concentration as 1 mg/mL and 0.25 mg/mL. The dosage of SEW2871 and VPC23019 used in this study was a slight modification to that reported by other authors [19–21]. In the consecutive 7 days after TBI, rats in TBI+Vehicle, TBI+SEW, TBI+VPC group, together with TBI+SEW+VPC group were treated, respectively, with an i.p. injection of DMSO (0.5 mL/kg/day), SEW2871 (1.0 mg/kg/day), VPC23019 (0.5 mg/kg/day), and a combination of SEW2871 and VPC23019.

For administration of the MEK/Erk inhibitor (Figures 1(a) and 1(b)), U0126 (Cell Signaling, 9903, Beverly, MA, USA) was dissolved in DMSO to a final concentration of 0.4 mg/mL. The dosage of U0126 followed that in previous studies with some modification [22, 23]. Rats in TBI+SEW+U0126 group were not only treated with i.p. injection of SEW2871 in the same way as described above, but also given to with intravenous (i.v.) injection of U0126 (0.2 mg/kg) via the tail vein twenty minutes before induction of TBI (while the rats were anesthetized). ERN, a bioactive compound from cruciferous vegetables, has been found to be an effective activator of MEK/Erk [24, 25]. Before administration of ERN (Figures 1(a) and 1(b)), its stock solution (Santa Cruz Biotechnology, sc-204741, Dallas, TX, USA) was diluted to a final concentration of 75 mmol/L with DMSO. Rats in TBI+VPC+ERN group received both VPC23019 treatment in the same way as described above and i.p. injection of ERN (12.5 mg/kg) twenty minutes before inducing TBI.

For administration of a thymidine analog to label the endogenous NSCs in hippocampus, BrdU (Sigma-Aldrich, B9285, St. Louis, MO, USA) was dissolved in 0.1 M sterile phosphate-buffered saline (PBS) to a final concentration of 10 mg/mL. On one hand, rats for the assessment of NSCs proliferation received three times i.p. injection of BrdU (100 mg/kg) with an 8-hour interval at the sixth day after TBI and were sacrificed for perfusion at the seventh day (Figure 1(a)). On the other hand, rats for the evaluation of NSCs neuronal differentiation were treated with an i.p. injection of BrdU (100 mg/kg) once per day from 1 to 7 days after TBI, and these rats were sacrificed for perfusion at 28 days after injury (Figure 1(b)).

### 2.4. Brain Tissue Preparation

At the scheduled time-points, rats were deeply anesthetized with sodium pentobarbital (60 mg/kg) and perfused with 50 mL of 0.9% saline through the left ventricle, and then by 4% paraformaldehyde in 0.1 M phosphate buffer saline (PBS) for 2 hours. The brains were removed and immersed in 4% paraformaldehyde in PBS (pH = 7.4) at 4°C overnight and then dehydrated by alcohol and embedded in paraffin. Coronal, 5 *μ*m thick brain sections containing the entire DG of hippocampus (from bregma −2.40 mm to −4.68 mm) were prepared by a microtome (Leica, Nussloch, Germany) and dried at 92°C overnight for immunofluorescence (IF). To assess the NSCs proliferation at 7 days after injury, a series of ten sections (120 *μ*m apart) from each rat brain were processed for BrdU/sex determining region Y-box 2 (SOX2) double-labeling IF. To assay the NSCs neuronal differentiation at 28 days after injury, another series of ten sections (120 *μ*m apart) parallel to the above ten from each brain were processed for BrdU/NeuN double-labeling IF.

### 2.5. IF

For DNA denaturation, brain sections were deparaffinized by alcohol and dimethylbenzene and then were incubated in the citric acid antigen retrieval buffer (pH = 6.0) at 95°C for 15 min. After that, the sections were incubated in PBS with 1% donkey serum albumin and 0.3% Triton X-100 at room temperature for 30 min to block nonspecific signals. For visualization of BrdU, SOX2, and NeuN, the sections were incubated in relevant primary antibodies, respectively, as follows: sheep anti-BrdU antibody (1 : 200, GeneTex, GTX21893, Irvine, CA, USA), rabbit anti-rat SOX2 antibody (1 : 500, GeneTex, GTX101507, Irvine, CA, USA), and mouse anti-rat NeuN antibody (1 : 500, Merck Millipore, MAB377, Billerica, MA, USA) in PBS overnight at 4°C. After three washes in PBS, the sections were incubated in relevant secondary antibodies as follows: Alexa fluor 594-labeled donkey anti-sheep IgG antibody (1 : 2000, Molecular Probes, A-11016, Eugene, OR, USA), Alexa fluor 488-labeled goat anti-rabbit IgG antibody (1 : 1000, Molecular Probes, A-11008, Eugene, OR, USA), and Alexa fluor 488-labeled goat anti-mouse IgG antibody (1 : 2000, Molecular Probes, A-11029, Eugene, OR, USA) in PBS for 1 hour at room temperature. Finally, the sections were washed 3 times in PBS, mounted with an anti-fade mounting medium containing 1, 4-Diazobicyclo (Electron Microscopy Sciences, CAT17895-01, Hatfield, PA, USA), and cover-slipped. All the procedures were performed in a manner that minimized light exposure to the tissue.

### 2.6. Microscopic Cell Counting

A confocal laser scanning microscope (FV1000, Olympus, Tokyo, Japan) with a FLUOVIEW image system (v.1.4a, Olympus, Tokyo, Japan) was used to capture the immunolabeled cells in hippocampus. BrdU^+^/SOX2^+^ cells and BrdU^+^/NeuN^+^ cells of five consecutive visual fields (24.41 *μ*m^2^ each, magnified by 400x) at DG in each section were counted; the value was averaged across the five visual fields and was thought to be the number of double-labeled cells for each section. Then, the average across the 10 sections was considered as the final number of double-labeled cells for each brain sample. The data were expressed as Mean ± SD. Images were assembled and labeled in Photoshop 7.0 (Adobe Systems, San Jose, CA, USA).

### 2.7. Western Blotting (WB)

Rats for the determination of S1PR1 protein were sacrificed at scheduled time-points and the brains were removed in the same way as described above. After split from brain on ice, the hippocampal tissues were homogenized and digested in a homogenizer with a lysis buffer (1% NP-40, 150 mM NaCl, 50 mM Tris (pH = 7.4), 1% Triton X-100, 0.5 mM EDTA, 1 mg/mL aprotinin, 1% deoxycholate, 10 mg/mL leupeptin, and 1 mM phenylmethylsulfonyl fluoride). The lysates were incubated on ice for 15 min and then centrifuged at 12,000 rpm for 30 min at 4°C. The protein concentration was examined by bicinchoninic acid Protein Assay kit (Beyotime, P0011, Shanghai, China). Then, sodium dodecyl sulfate (SDS) sample loading buffer was added in the supernatant and the mixture was boiled at 100°C for 5 min. Samples containing 40 *μ*g protein were resolved on 10% sodium dodecyl sulfate-polyacrylamide gel electrophoresis (SDS-PAGE) and electroblotted at 4°C for 50 minutes to nitrocellulose membrane. Following blocked by 5% skim milk in Tris-buffered saline solution containing 0.1% Tween-20 (TBST), the membranes were incubated overnight at 4°C with the following primary antibodies: rabbit anti-rat S1PR1 antibody (1 : 500, Prosci, 4809, Poway, CA, USA), rabbit anti-rat phosphate Erk (pErk) antibody (1 : 1000, Cell Signaling, 9101, Beverly, MA, USA), rabbit anti-rat total Erk (tErk) antibody (1 : 1000, Cell Signaling, 9102, Beverly, MA, USA), rabbit anti-rat phosphate MEK (pMEK) antibody (1 : 1000, Cell Signaling, 9154, Beverly, MA, USA), and rabbit anti-rat total MEK (tMEK) antibody (1 : 1000, Cell Signaling, 9126, Beverly, MA, USA). Rabbit anti-*β*-actin antibody (1 : 1500, Cwbiotech, CW0097, Beijing, China) was used as internal control for the concentration of immunoreactive proteins loaded.

After three washes in TBST, the membranes were incubated with horse radish peroxidase- (HRP-) conjugated goat anti-rabbit IgG antibody (1 : 20000, Cell Signaling Technology, 7074, Boston, MA, USA) for 1 hour at room temperature. The immunoreactive protein bands were visualized in Western Bright enhanced chemiluminescence reagents (K12045-d20, Advansta, Menlo Park, CA, USA). The immunoblots were analyzed by Gel-Pro Analyzer software (version 6.0, Media Cybernetics, Rockville, MD, USA). The ratio of S1PR1, pErk, tErk, pMEK, and tMEK to *β*-actin in gray scale (optical density value) was taken as the expression values, respectively.

### 2.8. Morris Water Maze (MWM) Test

At 24–28 days after injury, rats for spatial learning and memory test were performed with MWM trials [26]. A circular pool (160 cm diameter and 50 cm depth) filled with water (23 ± 2°C and 30 cm deep) was divided into four equal quadrants. There were 4 black plastic panels in different shape placed above the water surface serving as spatial cues for rat. All the objects in the experimental room remained at a constant position during testing process. A black circular platform (12 cm diameter) for rat escaping from water was submerged 1 cm under water surface in one of the four quadrants. The rat movements were recorded by a video tracking system placed 2 m above the pool center and analyzed by a data analysis system (DigBeh-MR, Shanghai Auspicious Software Technology Company Limited, China).

At 24–27 days after TBI, each rat performed four consecutive hidden platform trials per day in the maze with a trial-trial interval of 60 s. For each trial, rat was randomly placed into water facing the inside of the pool wall at one of the four quadrants and was freed to swim to find the hidden platform to escape from water. Each rat was given a maximum of 120 s to reach the platform and was allowed to remain on it for 15 s. If the rat failed to find the platform within 120 s, it was taken and placed onto the platform for 15 s. The time between rat being placed into water and reaching platform was recorded as escape latency. Average escape latency over the four consecutive days was regarded as the index of rat spatial learning capacity.

On 28 days after TBI, the platform was removed from the pool, and probe trails were conducted to assess spatial memory capacity of rats. Animals were allowed to swim freely for 120 s to find the previous platform which had been removed. “Platform crossing” referred to the times a rat swam over the location at which the platform was originally set in the hidden platform trials. Times of platform crossing and duration time spent in target quadrant were recorded. These two parameters were averaged and considered as the index of rat spatial memory capacity.

Following probe trails, visible platform trials were performed to assess swim capacity of each rat. A visible platform was placed above water surface in the pool, and rats were allowed swim freely to find the visible platform. Swim speed was recorded to assess rat motor activity in MWM test.

### 2.9. Statistical Analysis

Data was expressed as Mean ± SD and statistical analysis was processed by Graphpad Prisom software (v.6.01, Graphpad software, San Diego, CA, USA). Difference between groups was processed with one-way analysis of variance (ANOVA) and Tukey HSD* post hoc* test. The difference was considered statistically significant at *P* < 0.05.

## 3. Result

### 3.1. Expression of S1PR1 in Hippocampus Was Significantly Increased after TBI

**Figure 2:**
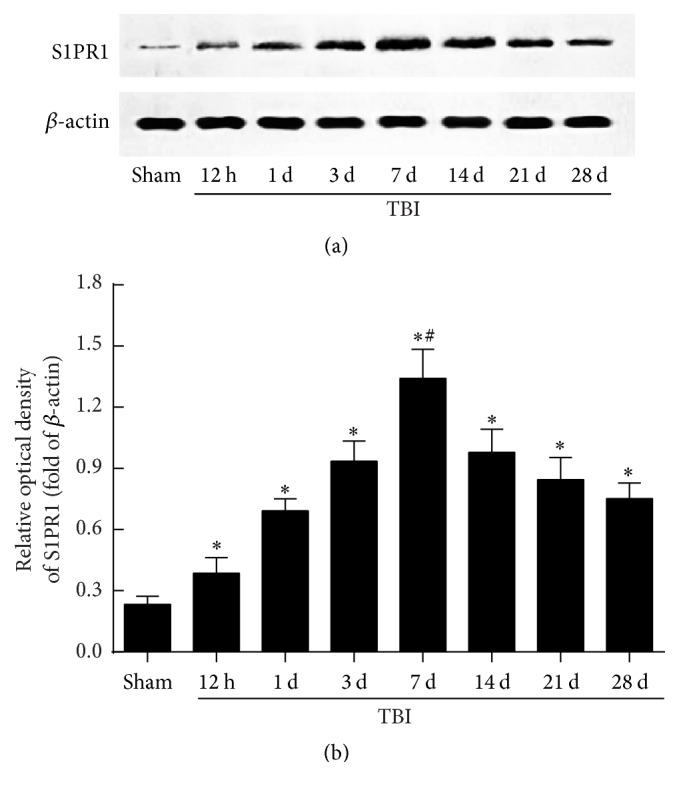
Hippocampal sphingosine 1-phosphate receptor 1 (S1PR1) expression increased markedly after TBI. (a) The level of S1PR1 protein in hippocampus was detected by western blotting (WB) at 12 hours, 1, 3, 7, 14, 21, and 28 days after trauma. (b) Quantitative analysis indicated that upregulated expression of hippocampal S1PR1 occurred from 12 hours, peaked at 7 days, and remained at a relatively higher level for at least 28 days after TBI (*n* = 3, sham group; *n* = 5 each time-point group of TBI). Further analysis revealed that there was a statistical difference between any two time-point groups after trauma. ^*∗*^ *P* < 0.05 versus sham group; ^#^ *P* < 0.05 versus other time-point groups of TBI.

The expression of S1PR1 in hippocampus at 12 hours, 1 day, 3 days, 7 days, 14 days, 21 days, and 28 days after TBI was determined by WB (Figure 2(a)). In the observed period, upregulated level of S1PR1 occurred from 12 hours after trauma, reached the peak at 7 days, and remained at a higher level than the sham at least 28 days (*P* < 0.05) (Figure 2(b)). Further analysis revealed that an obvious difference existed between any two time-point groups after trauma (*P* < 0.05) (Figure 2(b)). The data suggested that the hippocampal S1PR1 level was significantly increased after TBI.

### 3.2. S1PR1 Activation Enhanced NSCs Proliferation in Hippocampus after TBI

**Figure 3:**
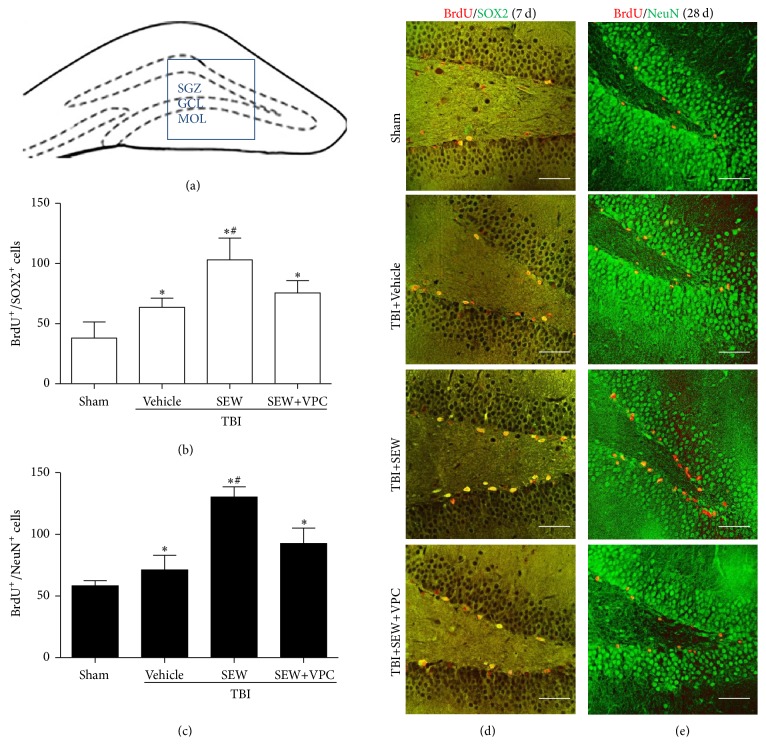
S1PR1 activation enhanced NSCs proliferation and neuronal differentiation in hippocampal dentate gyrus (DG) after TBI. (a) Coronal diagram of rat hippocampus, the subgranular zone, granular cells layer, and molecular layer of DG were, respectively, marked by SGZ, GCL, and MOL, the blue pane representing one of microscopic visual fields for counting immunolabeled cells of immunofluorescence (IF). (b) NSCs proliferation in SGZ at 7 days after TBI was assessed by BrdU/SOX2 double-labeling IF. Quantitation analysis showed that, relative to sham group (*n* = 3), brain injury induced more double-positive cells in TBI+Vehicle group (*n* = 6). Following the treatment of SEW2871, the number of BrdU^+^/SOX2^+^ cells further increased in TBI+SEW group (*n* = 6). Reversely, administration of VPC23019 resulted in a significant reduction of BrdU^+^/SOX2^+^ cells in TBI+SEW+VPC group (*n* = 6). (c) Neuronal differentiation of NSCs in SGZ at 28 days after TBI was assessed by BrdU/NeuN double-labeling IF. Statistical data indicated that BrdU^+^/NeuN^+^ cells increased in TBI+Vehicle group (*n* = 6) compared with sham group (*n* = 3). And the double-positive cells increased at even higher level after SEW2871 treatment in TBI+SEW group (*n* = 6). However, VPC23019 administration caused an evident decrease of BrdU^+^/NeuN^+^ cells in TBI+SEW+VPC group (*n* = 6). (d) and (e) Representative IF microphotographs of hippocampus immunolabeled with BrdU/SOX2 and BrdU/NeuN in all the above four groups at 7 days and 28 days after trauma. Scale bar: 50 *μ*m. ^*∗*^ *P* < 0.05 versus sham group; ^#^ *P* < 0.05 versus TBI+Vehicle group or TBI+SEW+VPC group.

The anatomic boundary of DG was identified as described previously [27] (Figure 3(a)). To investigate the influence of S1PR1 activation upon hippocampal neurogenesis after TBI, SEW2871 and/or VPC23019 was administrated to intervene the activity of S1PR1 in rats of corresponding groups. BrdU/SOX2 double-labeling IF was used to assess NSCs proliferation in DG at 7 days after injury (Figures 3(b) and 3(d)). As compared with sham group, the number of BrdU^+^ (red)/SOX2^+^ (green) cells in TBI+Vehicle group significantly increased at 7 days after TBI (*P* < 0.05). In addition, there were obviously more BrdU^+^/SOX2^+^ cells in TBI+SEW group than TBI+Vehicle group (*P* < 0.05). Statistical analysis showed that SEW2871 triggered an increase of NSCs proliferation by 62.31%. The results showed that TBI-induced NSCs proliferation in DG, and S1PR1 agonist further enhanced the TBI-induced NSCs proliferation. However, TBI+SEW+VPC group exhibited a decreased number of BrdU^+^/SOX2^+^ cells compared to that in TBI+SEW group (*P* < 0.05). The results suggested that application of S1PR1 antagonist reduced the effect of S1PR1 on NSCs proliferation. Taken together, all the above data indicated that S1PR1 activity was correlated with hippocampal NSCs proliferation after TBI.

### 3.3. S1PR1 Activation Promoted NSCs Differentiated into Neurons in Hippocampus after TBI

After understanding the effect of S1PR1 on NSCs proliferation in hippocampus, the potential association between S1PR1 expression and NSCs neuronal differentiation was then investigated. BrdU/NeuN double-labeling IF was performed to identify the hippocampal newly generated neurons, which were thought to be a main type of NSCs offspring for posttraumatic brain repair [3–5] (Figures 3(c) and 3(e)). Obviously, there were more BrdU^+^ (red)/NeuN^+^ (green) cells in TBI+Vehicle group than sham group at 28 days after trauma (*P* < 0.05). Moreover, the administration of S1PR1 agonist in the rats of TBI+SEW group markedly increased the number of BrdU^+^/NeuN^+^ cells compared to TBI+Vehicle group (*P* < 0.05). Nevertheless, the S1PR1-mediated neuronal differentiation of NSCs was abrogated by S1PR1 antagonist shown by a significant reduction of BrdU^+^/NeuN^+^ cells in TBI+SEW+VPC group (*P* < 0.05). The above data suggested that S1PR1 activation promoted the neuronal differentiation of NSCs in hippocampus after TBI.

### 3.4. MEK/Erk Signaling Pathway Was Activated in Response to the Upregulated S1PR1 after TBI

**Figure 4:**
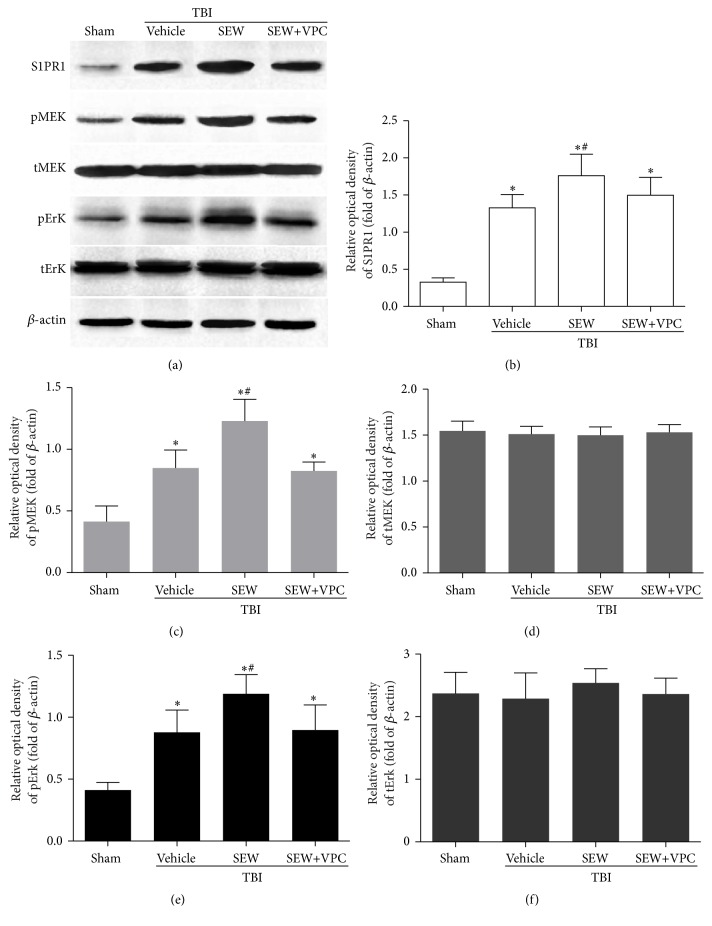
Activation of S1PR1 triggered MEK/Erk pathway phosphorylation in hippocampus at 7 days after TBI. (a) WB was performed to determine hippocampal S1PR1, pMEK, tMEK, pErk, and tErk expression in groups of sham, TBI+Vehicle, TBI+SEW, and TBI+SEW+VPC group (*n* = 3 in sham and *n* = 6 in other groups). (b, c, and e) The level of S1PR1, pMEK, and pErk significantly increased after trauma. SEW2871 induced further upregulation of S1PR1, pMEK, and pErk. However, the effect was attenuated by administration of VPC23019. (d, f) Neither S1PR1 agonism nor antagonism had effect on tMEK and tErk expression in hippocampus after TBI. ^*∗*^ *P* < 0.05 versus sham group; ^#^ *P* < 0.05 versus TBI+Vehicle group or TBI+SEW+VPC group.

It was well acknowledged that MEK/Erk cascade played a key role in multiple functions of neurogenesis, such as NSCs survival, proliferation, and differentiation. Hence, an investigation of the activity of MEK/Erk cascade in S1PR1-mediated NSCs proliferation and differentiation after TBI was performed by detecting S1PR1, pMEK, tMEK, pErk, and tErk expression in hippocampus with WB at day 7 after trauma (Figure 4(a)). As shown in Figures 4(b), 4(c), and 4(e), the levels of S1PR1, pMEK, and pErk in TBI+Vehicle group were dramatically increased compared with sham group (*P* < 0.05), and the TBI-induced S1PR1, pMEK, and pErk upregulation was further enhanced by the administration of S1PR1 agonist in TBI+SEW group (*P* < 0.05). However, this enhancement was attenuated by treatment with S1PR1 antagonist in TBI+SEW+VPC group (*P* < 0.05). Quantitation analysis revealed that there was no statistical difference in the level of tMEK and tErk between the four groups (*P* > 0.05) (Figures 4(d) and 4(f)). Taken together, the data revealed that the phosphorylation of MEK and Erk was activated in response to the increased S1PR1 after trauma. It was suggested that MEK/Erk signaling pathway might be the underlying downstream cascade to mediate S1PR1-associated hippocampal neurogenesis after TBI.

### 3.5. S1PR1 Induced Neurogenesis in Hippocampus Though MEK/Erk Cascade following TBI

**Figure 5:**
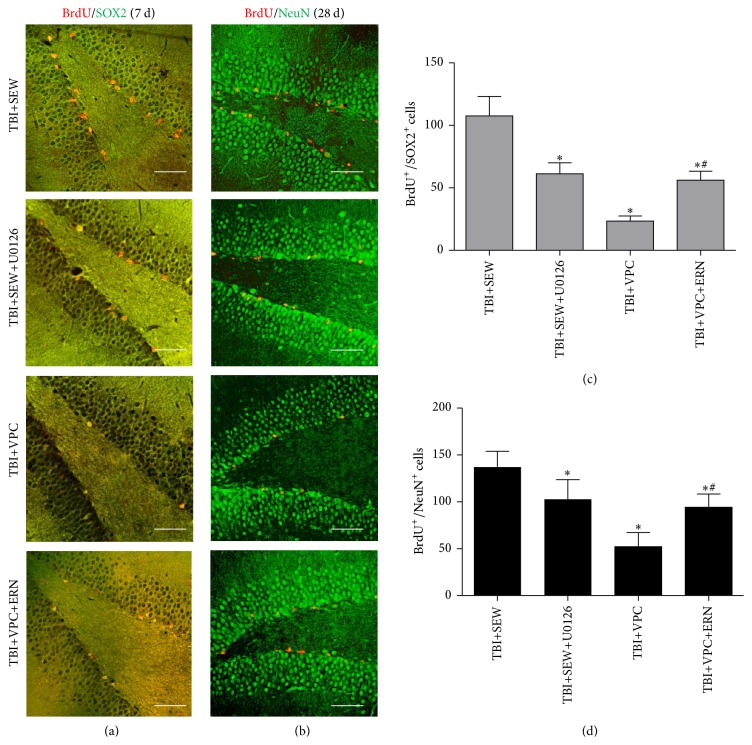
S1PR1-induced NSCs proliferation and neuronal differentiation in TBI rats were affected by MEK/Erk activity. (a, b) Representative IF microphotographs of hippocampus immunolabeled with BrdU/SOX2 and BrdU/NeuN in TBI+SEW, TBI+SEW+U0126, TBI+VPC, and TBI+VPC+ERN groups at 7 days and 28 days after trauma (*n* = 6 in each group). (c, d) Statistical analysis showed that, compared to TBI+SEW group, administration of U0126 significantly decreased the number of BrdU+/SOX2+ cells and BrdU+/NeuN+ cells in TBI+SEW+U0126 group. Reversely, the double-labeled cells in TBI+VPC+ERN group were significantly increased compared with TBI+VPC group. Scale bar: 50 *μ*m. ^*∗*^ *P* < 0.05 versus TBI+SEW group; ^#^ *P* < 0.05 versus TBI+VPC group.

To further demonstrate MEK/Erk signaling pathway contributing to S1PR1-associated neurogenesis after trauma, the effect of MEK/Erk inhibitor and activator upon NSCs proliferation at 7 days and NSCs neuronal differentiation at 28 days after TBI was studied (Figures 5(a) and 5(b)). On one hand, in comparison with TBI+SEW group, the number of BrdU^+^/SOX2^+^ cells and BrdU^+^/NeuN^+^ cells in TBI+SEW+U1026 group was decreased by 42.50% and 24.98%, respectively (*P* < 0.05) (Figures 5(c) and 5(d)); this data suggested that MEK/Erk inhibition mitigated the S1PR1-induced neurogenesis in hippocampus after TBI. On the other hand, the double-labeled cells in TBI+VPC+ERN group were significantly increased compared to TBI+VPC group at 7 and 28 days after trauma (*P* < 0.05) (Figures 5(c) and 5(d)), indicating that the detrimental effect of VPC231019 on NSCs proliferation and neuronal differentiation was rescued by ERN. Consequently, the above results implied that S1PR1 induced hippocampal neurogenesis via MEK/Erk pathway following TBI.

### 3.6. MEK/Erk Activity Contributed to S1PR1-Mediated Learning and Memory Performance in MWM Test

Since there was a close connection between hippocampal neurogenesis and cognitive function, the involvement of MEK/Erk signaling pathway in the S1PR1-mediated hippocampal learning and memory function was investigated by MWM test from 24 to 28 days following TBI.

**Figure 6:**
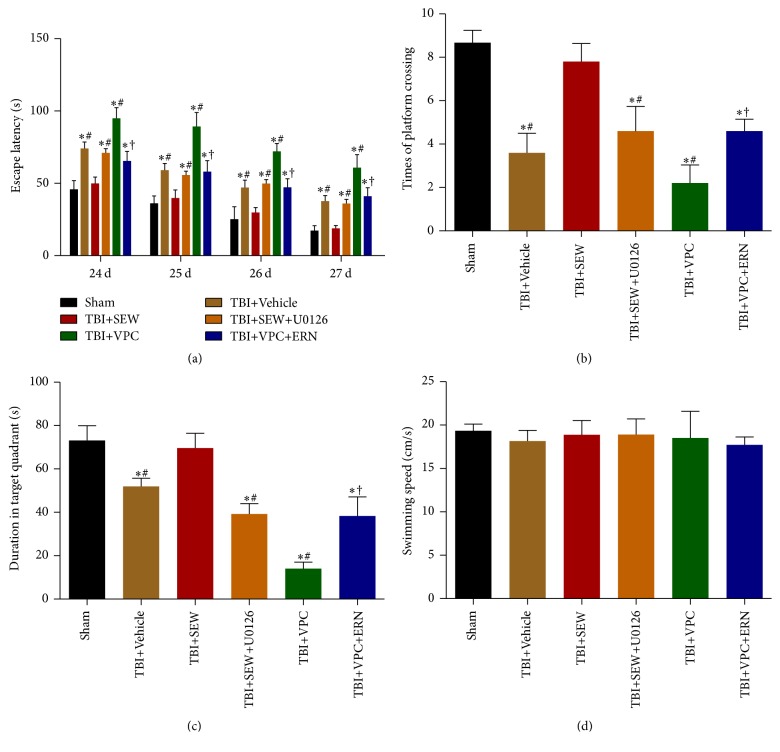
Cognitive function of rats in sham, TBI+Vehicle, TBI+SEW, TBI+SEW+U0126, TBI+VPC, and TBI+VPC+ERN group (*n* = 3 in sham and *n* = 5 in other groups) were evaluated by Morris water maze (MWM) test. (a) Escape latency in hidden platform trial exhibited a gradual reduction tendency from 24 to 27 days after trauma. Daily escape latency of TBI+Vehicle group was longer than that of sham group. Use of S1PR1 agonist in TBI+SEW group significantly shorten the latency, but the effect was eliminated in TBI+SEW+U0126 group. In addition, the escape latency of TBI+VPC+ERN group decreased compared with TBI+VPC group. (b, c) Platform crossing times and target quadrant duration in probe trial revealed that, relative to sham group, TBI+Vehicle group presented less times and shorter duration at 28 days after TBI. S1PR1 activation significantly increased the two indexes of TBI+SEW group, but the favorable effect was blocked by U0126 treatment in TBI+SEW+U0126 group. Moreover, the times of platform crossing and duration in target quadrant of TBI+VPC group were lower than those of TBI+VPC+ERN group. (d) Rat swimming speed of the six groups did not show any statistical difference. ^*∗*^ *P* < 0.05 versus sham group; ^#^ *P* < 0.05 versus TBI+SEW group, ^†^ *P* < 0.05 versus TBI+VPC group.

In hidden platform trials, a gradual decrease in rat escape latency across 24, 25, 26, and 27 days after trauma was presented in Figure 6(a). It was clear that the daily escape latency of TBI+Vehicle group was significantly longer than sham group (*P* < 0.05), indicating that rat learning function was impaired by TBI. TBI+SEW group showed a much shorter latency than TBI+Vehicle group (*P* < 0.05), suggesting that administration of SEW2871 improved the impaired learning performance after TBI. In addition, the rats in TBI+SEW+U0126 group spent longer escape latency searching the hidden platform compared with rats in TBI+SEW group (*P* < 0.05). Moreover, the latency of TBI+VPC+ERN group was obviously less than that of TBI+VPC group (*P* < 0.05). These data implied that S1PR1-mediated learning function was affected by MEK/Erk activity after TBI.

In probe trails, platform crossing times and duration spent in target quadrant at 28 days after injury were shown in Figures 6(b) and 6(c), respectively. These two indexes of TBI+Vehicle group were less than those of sham group (*P* < 0.05). Compared with TBI+Vehicle group, rats in TBI+SEW group exhibited better performance in probe trails (*P* < 0.05). In addition, TBI+SEW+U0126 group presented evidently decreased times and duration compared to TBI+SEW group (*P* < 0.05). Furthermore, the platform crossing times and target quadrant duration of TBI+VPC+ERN group were significant below those of TBI+SEW group (*P* < 0.05). Taken together, the above data indicated that S1PR1 activation improved memory impairment caused by TBI via MEK/Erk pathway.

In visible platform trials, all rats swam and found the platform normally. The average swimming speed of the rats in each group was 19.34 ± 0.77 cm/s, 18.15 ± 1.24 cm/s, 18.88 ± 1.64 cm/s, 18.90 ± 1.82 cm/s, 18.51 ± 3.08 cm/s, and 17.73 ± 0.90 cm/s, respectively (Figure 6(d)); the difference between groups did not reach a statistically significant level (*P* > 0.05). This data indicated that rat swimming speed of each group was at the same level, suggesting that the swimming speed exerted no impact on the above results in MWM test. In addition, the statistic difference between TBI+SEW group and sham group in the above trails was not observed (*P* > 0.05) (Figure 6), further implying that activation of S1PR1 facilitated learning and memory recovery after TBI.

## 4. Discussion

Increasing evidence suggests that brain injury is capable of stimulating hippocampal NSCs to divide and generate new neurons for repairing [3, 7]. However, the level of hippocampal neurogenesis after TBI is inadequate to meet the needs of injury repairing and neurological function recovery [3, 6]. Thus, it is valuable to effectively boost neurogenesis for traumatic brain rehabilitation by uncovering the potential molecule and the underlying mechanisms. The present study pointed out that activation of S1PR1 improved rat hippocampal neurogenesis via MEK/Erk signaling pathway after TBI. Initially, we confirmed that TBI could trigger NSCs proliferation and neuronal differentiation in hippocampus. Afterwards, we found that S1PR1 activation further promoted the hippocampal neurogenesis induced by TBI, and the enhancement could be attenuated by the antagonism of S1PR1. Moreover, it was revealed that MEK and Erk phosphorylation was activated in the S1PR1-mediated neurogenesis after TBI. Inhibition of MEK/Erk abolished the neurogenesis improved by S1PR1 activation. Reversely, the unfavorable effect of S1PR1 antagonism on posttraumatic neurogenesis and cognition recovery was rescued by the activation of MEK/Erk.

Recent studies demonstrated that S1PR1 was highly expressed in NSCs, and S1PR1 activation was involved in NSCs migration induced by high S1P concentration* in vitro* [14, 15]. In particular, S1PR1 contributed much to the migration of transplanted NSCs toward the injured site of spinal cord [15]. The study by Harada et al. suggested that S1P was essential for NSCs proliferation and morphological changes during brain development, but the subtype of S1PRs family contributed to S1P-induced NSCs activity was not clearly defined [16]. According to [35S]GTP*γ*S autoradiography map of embryonic brain, the authors speculated that S1P1 might be the subtype receptor mediating the effect of S1P on NSCs proliferation and morphological changes [16]. In addition, it has been documented that S1PR1 is expressed abundantly in both hippocampus and SVZ, which are two specific regions responsible for neurogenesis throughout life in rodent brain [13, 28]. However, the effect of S1PR1 on endogenous NSCs properties in hippocampus after CNS trauma remains poorly understood.

In this study, it was shown that S1PR1 expression in hippocampus increased significantly with a maximal level at 7 days and remained at a higher level than sham at 28 days after TBI. Hippocampal NSCs proliferation after TBI also peaked at 7 days after trauma, as demonstrated in numerous previous studies [3, 29, 30]. Accordingly, it can be considered that TBI-induced neurogenesis might correlate with the upregulation of S1PR1 protein in hippocampus. More importantly, through i.p. administration of S1PR1 agonist in rats, we found the activated S1PR1 could further promote NSCs proliferation and neuronal differentiation in hippocampus after TBI, and the effect was abolished by antagonism of S1PR1. These results indicated that S1PR1 activation facilitated posttraumatic neurogenesis in hippocampus, which might provide a clue to develop new strategies for brain repairing and functional recovery after TBI.

In several previous studies, it was well acknowledged that MEK/Erk cascade was critical for the activation of transcription factors and genes to regulate NSCs proliferation and differentiation, as well as the maturation of NSCs-derived neuronal cells [18, 31, 32]. Recently, growing researches have demonstrated an involvement of MEK/Erk cascade in neurogenesis induced by diverse growth factors, such as neurotrophin-3 and vascular endothelial growth factor (VEGF) [33–35]. Particularly, Ge and his colleagues found that MEK/Erk pathway was required for the neuronal differentiation but not for the oligodentrocytic differentiation of NSCs induced by poly-L-ornithine* in vitro* [36]. Furthermore, MEK/Erk pathway has been reported to play protective roles for neurons survival in DG [17, 32] and also contribute to the antiapoptotic effect of S1PR1 on neurons in injured cortex after cerebral ischemia [19].

In this study, it was observed that the levels of hippocampal S1PR1, pMEK, and pErk in TBI group were higher at 7 days after trauma compared with sham group, and that activation of S1PR1 by SEW2871 further increased the hippocampal pMEK and pErk expression. Consistently, this effect could be eliminated by antagonism of S1PR1. The results implied that a potential association might exist between S1PR1 activation and MEK/Erk cascade phosphorylation in hippocampal neurogenesis after TBI. In addition to our findings, other studies have shown that the upregulated expression of pErk at 8 hours, 1 day, and even 14 days after CNS injury also played a positive role in hippocampal neurogenesis [31, 32, 37]. Accordingly, these evidences collectively provided an insight into the regulatory role of MEK/Erk signaling in neurogenesis after TBI.

Moreover, MEK/Erk pathway has been implicated in S1P-associated survival and proliferation of human embryonic stem cells, though the subtype of S1PRs accounting for the process remains to be determined [38]. In our study, following administration of MEK/Erk inhibitor in TBI rats, a significant decrease of the proliferation and neuronal differentiation of NSCs was observed in hippocampus, indicating that MEK/Erk signaling pathway was indeed essential for S1PR1-mediated hippocampal neurogenesis after TBI. However, the underlying mechanisms in which S1PR1 triggers the phosphorylation of MEK and Erk in posttraumatic neurogenesis require further investigation.

It was noteworthy that a lot of past studies highlighted the influence of MEK/Erk pathway on NSCs properties such as proliferation and survival, whereas the effect of MEK/Erk on NSCs differentiation in hippocampal neurogenesis after TBI was not fully elaborated [16, 18, 35]. In the present study, besides probing the involvement of MEK/Erk signaling in S1PR1-associated NSCs proliferation, we extended our investigation on the role of MEK/Erk cascade in S1PR1-associated neuronal differentiation of NSCs at 28 days after TBI. The data showed that S1PR1 agonist via i.p. injection significantly increased the number of NSCs-derived newborn neurons in DG. And inhibition of MEK/Erk signaling resulted in a marked suppression in the S1PR1-induced neuronal differentiation of NSCs. Moreover, activation of MEK/Erk restored the diminished hippocampal neurogenesis caused by S1PR1 antagonist after TBI. Therefore, it can be inferred that activation of S1PR1 enhances hippocampal NSCs differentiate into neurons though MEK/Erk pathway after TBI. However, the precise downstream cascade of MEK/Erk responsible for S1PR1-meidated NSCs differentiation remains elusive. Most recently, quite a few evidences pointed out that cAMP-response element binding protein (CREB) was the principal downstream target of MEK/Erk signaling to regulate hippocampal plasticity [35, 37, 39]. Our research team is on the way to explore the presumed role of MEK/Erk/CREB cascade in multidirectional differentiation of NSCs induced by S1PR1.

As the paramount structure of limbic system, hippocampus links closely to cognitive and behavioral formation, which is easily impaired by brain injury [40]. Accumulative evidence supported that the newborn neurons in the posttraumatic neurogenesis could functionally integrate into the injured neural network and facilitate hippocampus-dependent neurocognitive recovery [6, 7, 41]. It has been reported that S1PR1 activation was in a position to ameliorate hippocampal damage and spatial memory deficits of Alzheimer's disease rats [42]. In addition, a recent study revealed that a known S1PRs agonist, FTY720, could ameliorate sevoflurane-induced neurocognitive impairment by distinctively binding to S1PR1 and activating the downstream pathway [43]. These findings indicated that the activity of S1PR1 indeed correlated with hippocampus-dependent cognitive function in a variety of neurological diseases, such as neurotoxicity and neurodegeneration [42, 43]. Here, we found that the learning and memory performance was improved by S1PR1 activation in neurotrauma, inhibition of MEK/Erk cascade worsened the learning and memory performance elicited by S1PR1 agonist after TBI. Furthermore, the adverse effect of S1PR1 antagonism on neurocognition after TBI was meliorated by MEK/Erk activation. It indicates that MEK/Erk signaling pathway is critical to S1PR1-mediated hippocampal neurogenesis for posttraumatic neurocognitive recovery.

## 5. Conclusion

In summary, our results indicated that S1PR1 produced a favorable effect of activation on NSCs proliferation and neuronal differentiation in hippocampus after TBI, and MEK/Erk signaling pathway played a key role in the S1PR1-mediated hippocampal neurogenesis. In the future, a deep exploration into the underlying mechanism will be required before S1PR1 was considered as a promising target for strategies to promote hippocampal neurogenesis for TBI repairing.
